# High quantities: Evaluating the association between cannabis use and propofol anesthesia during endoscopy

**DOI:** 10.1371/journal.pone.0248062

**Published:** 2021-03-04

**Authors:** Ngozi Imasogie, Rhiannon V. Rose, Aze Wilson

**Affiliations:** 1 Department of Anesthesia, Yorkton Regional Centre, Yorkton, SK, Canada; 2 Department of Epidemiology and Biostatistics, Western University, London, ON, Canada; 3 Division of Clinical Pharmacology, Department of Medicine, Western University, London, ON, Canada; 4 Division of Gastroenterology, Department of Medicine, Western University, London, ON, Canada; University of the West of Scotland, UNITED KINGDOM

## Abstract

**Background:**

Endoscopy under propofol sedation has become a routine procedure. Given the number of Canadians undergoing an endoscopy annually, as well as the pervasive use of cannabis by many patients, understanding the effect of cannabis use on the propofol dose at endoscopy is highly relevant. We aimed to evaluate the association between cannabis exposure and the propofol dose needed to achieve adequate sedation at endoscopy.

**Methods:**

A case-control study of individuals undergoing endoscopy was conducted at a single outpatient endoscopy clinic in London, Ontario between 2014 and 2017. Cases included all individuals with any self-reported cannabis exposure, while controls included all individuals without any self-reported history of cannabis use. Dose of propofol administered by a single anesthetist was collected on each subject as well as additional demographic and procedure-related covariates.

**Results:**

Three hundred and eighteen participants were included (cases, n = 151; controls, n = 167). Cannabis exposure was associated with an increase in propofol dose (cases 0.33 mg/kg/minute ±0.24; controls, 0.18 mg/kg/minute ±0.11; p<0.0001). Cannabis exposure remained an independent predictor of propofol dose on multivariate linear regression accounting for other important covariates (p<0.0001). Daily cannabis users required a higher propofol dose than weekly or monthly users. Three procedural sedation-related complications occurred in the cannabis-exposed group, while none occurred in the unexposed group.

**Conclusion:**

Our data suggest that cannabis use is significantly associated with the quantity of propofol needed for sedation at endoscopy. Further study is needed to better understand the molecular basis for this possible drug-drug interaction.

## Introduction

Cannabis, commonly known as marijuana, is one of the most frequently used substances worldwide [1]. According to the United Nations Office on Drug and Crime World Drug Report, the highest-prevalence regions include North America, Australia and parts of Africa, with the highest rates reported amongst individuals 15 to 64 years of age. Acute use of cannabis is linked to impairments in cognitive skills and psychomotor function [2]. The long-term effects of cannabis use on patient outcomes are not well defined, though it has been linked to persistent deficits in executive functioning, memory, and concentration [2]. Like any other drug, it has the potential to interact with and affect the efficacy of other concurrently-administered medications. Its effect on anesthesia has not been well-documented beyond small prospective studies, case reports and anecdotal accounts [3–7].

Endoscopy under propofol sedation has become a routine procedure for colorectal cancer screening as well as for the diagnosis and management of various chronic gastrointestinal diseases such as inflammatory bowel disease and celiac disease. In this setting, initial doses of propofol used fall below what would be required for induction of general anesthesia for a surgical procedure requiring intubation and respiratory support. High doses of propofol are linked to hypotension, bradycardia, respiratory depression, and vomiting.

Given the number of individuals undergoing an endoscopic procedure annually as well as the pervasive use of cannabis by many patients, understanding the effect of cannabis use on endoscopy-related sedation is highly relevant. We aimed to evaluate the effect of cannabis exposure on the propofol dose needed to achieve adequate sedation at endoscopy.

## Materials and methods

A case-control study of individuals undergoing endoscopy (colonoscopy and/or esophagogastroduodenoscopy, EGD) was conducted at a single outpatient endoscopy clinic in London, Ontario between 2014 and 2017. Cases were defined as individuals with any duration of self-reported inhaled cannabis exposure. Cases were further sub-divided by frequency of cannabis use into the following groups for further analysis: daily users (cannabis use at least 4 out of 7 days per week), weekly users (cannabis use 1–2 days per week at least 3 weeks per month), monthly users (cannabis use up to 1–2 times per month at least 9 months per year) and occasional users (cannabis use less than once every 2 months). Controls were defined as individuals who had no past or present history of cannabis use. Eligible subjects were greater than 17 years of age and undergoing an endoscopic procedure under propofol sedation without additional procedural co-medication. Exclusion criteria included any history of renal or hepatic impairment, pregnancy, or an incomplete medical history. The dose of propofol required to achieve and maintain adequate sedation in milligrams (mg) was recorded at the time of the procedure; all doses were administered by a single anesthesiologist as incremental boluses over the course of the procedure. The Modified Observer’s Assessment of Alertness/Sedation (MOAA/S) scale was used to assess the depth of sedation with a score 1 or 0 defined as adequate sedation [8]. Additional demographic and procedure-related covariates were collected including, age, sex, weight in kilograms (kg), past medical history including the presence or absence of chronic and respiratory disease were noted in addition to drug use (including narcotics, anxiolytics, antidepressants), alcohol exposure, procedure type, duration, and presence of procedural sedation-related complications. A standardized data collection form was used by the study team to abstract data from patients’ charts. Data abstraction was performed by two experienced reviewers. All charts were reviewed by a second reviewer for accuracy and discrepancies were resolved by consensus. Training sessions on the study protocol and use of the data abstraction form were provided. Pilot testing of the data collection forms was performed by reviewers in a small subset of consented participants to ensure the reliability and validity of the data collection forms.

The primary endpoint was dose of propofol per kg of weight per minute of procedure. Secondary outcomes included dose of propofol by frequency of cannabis exposure (daily, weekly, monthly, occasional, never), and procedural sedation-related complications. The study protocol was approved by the Western University Health Sciences Research Ethics Board (109150). The requirement for written informed consent was waived by the Research Ethics Board. Data were analyzed anonymously.

All data were analyzed using Graphpad Prism or R statistical software. Descriptive statistics were used to summarize demographic data. A Student’s t-test was used to assess differences between cases and controls for all demographic and clinical variables. A Student’s t-test was used to assess differences in the mean propofol dose per kg per minute of procedure between cases and controls. A Fisher’s exact test was used to evaluate the rate of procedural sedation-related complications. A multivariable linear regression analysis was used to estimate the degree of association between cannabis exposure and propofol dose in the presence of other covariates. Covariates used in the final model included: age, sex, weight, American Society of Anesthesiologists Score (ASA), presence or absence of chronic pain and/or a respiratory disease, smoking status, alcohol exposure, narcotic and/or benzodiazepine use, and procedure duration. For all analyses, a p-value of <0.05 was considered significant.

## Results and discussion

**Fig 1:**
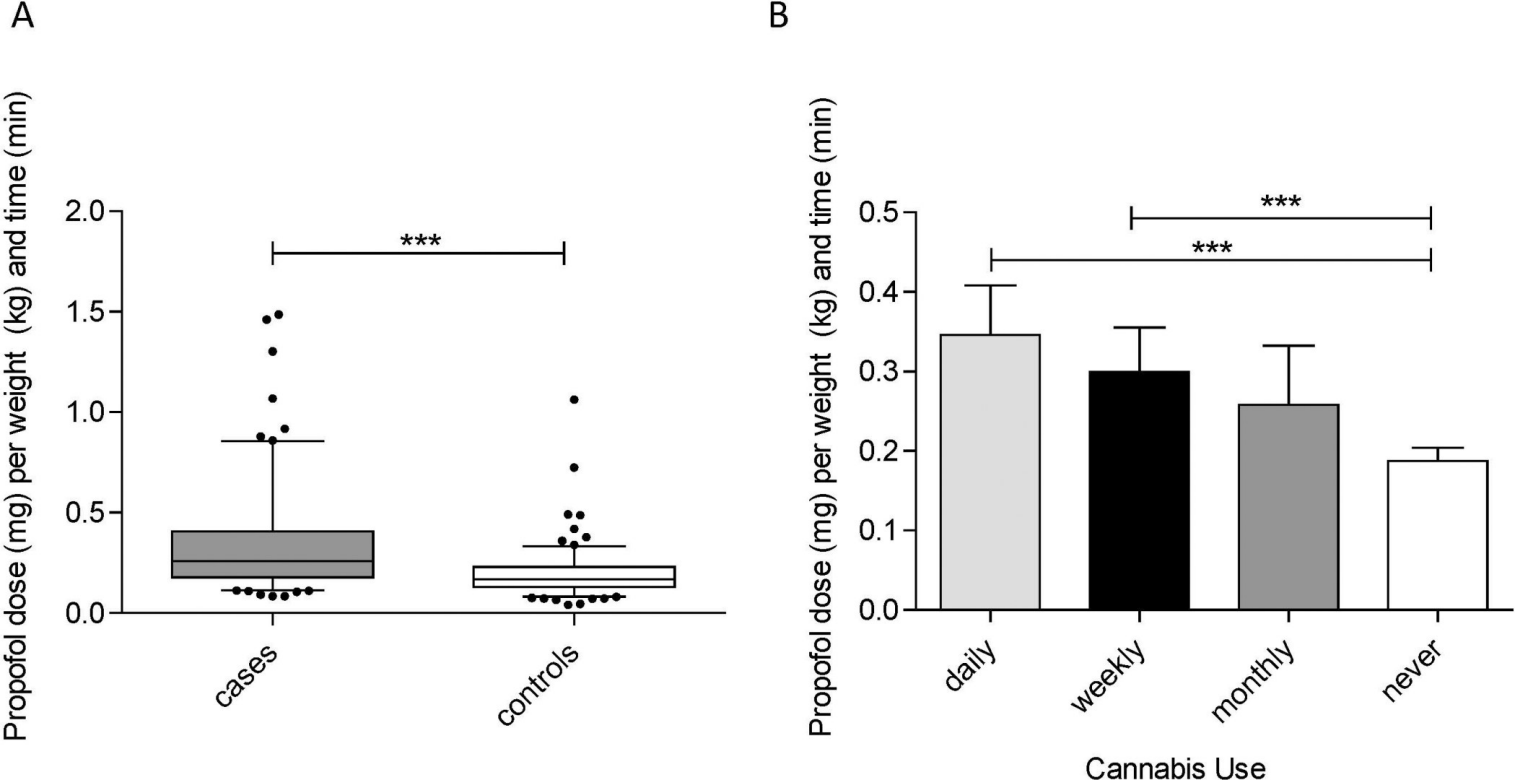
Mean propofol dose per unit of weight in kilograms and time in minutes for cases and controls (A) as well as sub-divided by frequency of cannabis use (B). Box plot outlines represent the 25^th^ and 75^th^ percentile values (A). Error bars (A and B) represent the 95% confidence interval. *p<0.05, **p<0.01, ***p<0.001.

Baseline characteristics for all study subjects are presented in Table 1. Charts for 340 individuals undergoing endoscopy (EGD or colonoscopy) under propofol sedation were screened. One hundred and fifty-one cases (cannabis-exposed) and 167 controls (cannabis-unexposed) were included in the final analyses. Twenty-two individuals were excluded due to incomplete study-related medical data or renal insufficiency. Cannabis users were more likely to be younger, male and concurrent cigarette smokers. The majority of cannabis users (53.0%) were daily users. Cannabis exposure was associated with a significantly higher propofol dose to achieve adequate sedation as defined by the MOAA/S scale compared to those without cannabis exposure (cases 0.33 mg/kg/minute ±0.24; controls, 0.18 mg/kg/minute ±0.11; p<0.0001) (Fig 1A). Daily cannabis users required a higher dose of propofol than weekly or monthly users (Fig 1B).

**Table 1:** Population demographics.

Characteristics	Cases (n = 151)	Controls (n = 167)	p-value
Age, years (mean, range)	43.76 (18–71)	53.8 (23–88)	p<0.0001
Female Sex (%)	46 (30.4)	101 (60.4)	p<0.0001
Weight, kg (mean ± std)	82.9±23.4	77.4±17.2	p<0.05
ASA score (mean ± std)	2.25±0.88)	2.24±0.82	ns
Smoking history	104 (68.9)	69 (41.3)	p<0.05
Narcotic use (%)	27 (17.9)	16 (9.6)	ns
Respiratory disease^¥^ (%)	39 (25.8)	28 (16.8)	ns
Anxiolytic/antidepressant use (%)	47 (31.1)	34 (20.4)	ns
Cannabis use (%)			
Daily	80 (53.0)	-	-
Weekly	36 (23.8)	-	-
Monthly	13 (8.6)	-	-
Occasionally*	19 (12.6)	-	-
Procedure type (%)			
Colonoscopy	80 (53.0)	114 (68.2)	ns
EGD	50 (33.1)	34 (20.4)	ns
EGD & Colonoscopy	21 (13.9)	19 (11.4)	ns
Procedure Time, min (mean ± std)	16.7±10.9	18.3±9.6	ns
Propofol dose, mg (mean ± std)	310.9±113.1	220.8±91.8	p<0.0001

^¥^Respiratory disease is defined as a self-reported history of chronic obstructive pulmonary disease, asthma or obstructive sleep apnea.

*Occasionally is defined as using cannabis less than once every 2 months.

Standard deviation, std; American Society of Anesthesiologists score, ASA score; kilogram, kg; minutes, min; milligram, mg; not significant, ns.

Cannabis exposure remained an independent predictor of propofol dose on multivariable linear regression analysis (F (15,298) = 33.64, p<0.0001) with the model accounting for 61% of the propofol dose variability (Table 2). Study subject propofol dose increased by 75.98mg with daily cannabis exposure (p<0.0001). Other significant covariates adjusted for in the model were age (p<0.0001), presence of a respiratory disease (one of asthma, obstructive sleep apnea or chronic obstructive pulmonary disease, p = 0.024), weight (p<0.0001) and duration of procedure (p<0.0001). Three procedural sedation-related complications (increased oxygen requirements necessitating bag-mask intervention with oral airway) occurred in the cannabis-exposed group, while none occurred in the unexposed group (p>0.05).

**Table 2:** Multiple linear regression model for the degree of association between cannabis exposure and propofol dose (n = 318, adjusted R^2^ = 0.61).

Variable	β-coefficients	Lower 95% CI	Upper 95% CI	p-value
Intercept	116.08	63.66596	168.5035	**<0.0001**
Daily Cannabis exposure	75.98	53.32552	98.64701	**<0.0001**
Age (years)	-2.10	-2.7401	-1.47107	**<0.0001**
Male Sex	4.23	-13.6917	22.1659	0.64
Weight (kg)	1.09	0.651074	1.540829	**<0.0001**
ASA score>1	-1.47	-24.8945	21.95445	0.90
History of respiratory disease	23.19	2.589676	43.8051	**0.024**
History of chronic pain	11.58	-17.9667	41.14491	0.44
Smoking history	13.01	-3.95242	29.98339	0.13
Alcohol (>2 drinks per week)	0.91	-0.06237	1.885968	0.06
Narcotic and or benzodiazepine use	3.15	-25.8116	32.1133	0.83
Procedure duration (minutes)	6.29	5.273378	7.314243	**<0.0001**

Confidence interval, CI; American Society of Anesthesiologists Score, ASA; kilogram,kg.

Given the pervasive use of cannabis amongst patients, determining the impact of cannabis exposure on procedure-related sedation is extremely important. There are a limited number of small studies and case reports suggesting a greater risk of complication related to anesthesia or higher tolerance to procedural-related sedation amongst cannabis users [3,6]. Our data suggest that cannabis use is significantly associated with the quantity of propofol needed for sedation at endoscopy. Higher doses of propofol were needed amongst cannabis users compared to non-users, with the highest doses needed by daily cannabis users. Unsurprisingly, weight, age, procedure duration and chronic respiratory disease were also significantly associated with final propofol dose.

Cannabis exposure was not associated with an increased rate of procedural sedation-related complications; however, the small study sample size described may have limited the ability to detect differences between the groups. Other study limitations included a lack of formal inter-rater variability assessment, though the use of a standardized data collection tool as well as experience reviewers were utilized to minimize any biases introduced due the presence of multiple observers. Additionally, there was an inability to control for variations in cannabis tetrahydrocannabinol content amongst the cannabis-exposed population. The study populations were also significantly different across a number of variables, including age, sex, and weight. This may reflect inherent differences between those who use cannabis and those who do not, which could also affect a patient’s baseline required propofol dose, independent of cannabis usage. These differences were accounted for with multivariable analysis. Cannabis exposure remained an independent predictor of propofol dose when these covariates were adjusted for. Additionally, this association may not be causal. Further study is needed to better understand the molecular basis for this possible drug-drug interaction. Though disputed by some [9], current *in vivo* models suggest that propofol may impart a portion of its sedative effect via the endocannabinoid system [10,11]. Plausible hypotheses regarding a propofol-cannabis interaction include down-regulation of the cannabinoid (CB)-1 receptor in chronic cannabis users versus partial agonism/antagonism at the CB-1 receptor by other phytocannabinoids in marijuana products that may compete with propofol, increasing the required dose [12]. It is additionally unclear if these effects are mediated via tetrahydrocannabinol or some other compound. Inhibition of propofol due to other unrecognized inhibitors contained within the marijuana product may potentially occur.

## Conclusions

Ultimately, cannabis exposure is significantly associated with propofol dose at endoscopy. Prospective evaluation and mechanistic studies are needed to further define this relationship.

## Supporting information

S1 Data Minimum data set in CSV format. (CSV)

## References

[1] UNODC. World Drug Report. Vienna: United Nations Office on Drug and Crime; 2015.

[2] Crean RD, Crane NA, Mason BJ. An evidence based review of acute and long-term effects of cannabis use on executive cognitive functions. J Addict Med. 2011;5(1):1. 10.1097/ADM.0b013e31820c23fa 21321675 PMC3037578

[3] Georg R, Götz B, Arlt F, Heymann Cv. Cannabis consumption before surgery may be associated with increased tolerance of anesthetic drugs: A case report. International Journal of Case Reports and Images (IJCRI). 2015;6(7):436–9.

[4] Flisberg P, Paech M, Shah T, Ledowski T, Kurowski I, Parsons R. Induction dose of propofol in patients using cannabis. European Journal of Anaesthesiology (EJA). 2009;26(3):192–5. 10.1097/EJA.0b013e328319be59 00003643-200903000-00003. 19237981

[5] Coté GA, Hovis RM, Ansstas MA, Waldbaum L, Azar RR, Early DS, et al. Incidence of sedation-related complications with propofol use during advanced endoscopic procedures. Clin Gastroenterol Hepatol. 2010;8(2):137–42. 10.1016/j.cgh.2009.07.008 19607937

[6] Symons IE. Cannabis smoking and anaesthesia. Anaesthesia. 2002;57(11):1142–3. 10.1046/j.1365-2044.2002.288312.x 12392470

[7] Twardowski MA, Link MM, Twardowski NM. Effects of Cannabis Use on Sedation Requirements for Endoscopic Procedures. J Am Osteopath Assoc. 2019. Epub 2019/04/16. 10.7556/jaoa.2019.052 . 30985870

[8] Sheahan CG, Mathews DM. Monitoring and delivery of sedation. BJA: British Journal of Anaesthesia. 2014;113(suppl_2):ii37–ii47. 10.1093/bja/aeu378 25498581

[9] Hauer D, Ratano P, Morena M, Scaccianoce S, Briegel I, Palmery M, et al. Propofol enhances memory formation via an interaction with the endocannabinoid system. Anesthesiology. 2011;114(6):1380–8. Epub 2011/05/03. 10.1097/ALN.0b013e31821c120e . 21532463

[10] Brand P, Meybohm P, Scholz J, Werner C, Bein B. Cannabinoid 1 receptor antagonist reduces THC/propofol interaction: 9AP4-9. European Journal of Anaesthesiology (EJA). 2007;24:117. 00003643-200706001-00435.

[11] Patel S, Wohlfeil ER, Rademacher DJ, Carrier EJ, Perry LJ, Kundu A, et al. The general anesthetic propofol increases brain N-arachidonylethanolamine (anandamide) content and inhibits fatty acid amide hydrolase. Br J Pharmacol. 2003;139(5):1005–13. Epub 2003/07/04. 10.1038/sj.bjp.0705334 12839875 PMC1573928

[12] Morales P, Hurst DP, Reggio PH. Molecular Targets of the Phytocannabinoids: A Complex Picture. Progress in the chemistry of organic natural products. 2017;103:103–31. 10.1007/978-3-319-45541-9_4 . 28120232 PMC5345356

